# Using deep learning to analyze the psychological effects of COVID-19

**DOI:** 10.3389/fpsyg.2023.962854

**Published:** 2023-08-14

**Authors:** Monira Abdulrahman Almeqren, Latifah Almuqren, Fatimah Alhayan, Alexandra I. Cristea, Diane Pennington

**Affiliations:** ^1^Education College, Princess Nourah Bint Abdulrahman University, Riyadh, Saudi Arabia; ^2^Department of Information Systems, College of Computer and Information Sciences, Princess Nourah bint Abdulrahman University, Riyadh, Saudi Arabia; ^3^Department of Computer Science, University of Durham, Durham, United Kingdom; ^4^Department of Computer Science, University of Strathclyde, Glasgow, United Kingdom

**Keywords:** sentiment analysis, anxiety, Arabic, COVID-19, deep learning

## Abstract

**Problem:**

Sentiment Analysis (SA) automates the classification of the sentiment of people’s attitudes, feelings or reviews employing natural language processing (NLP) and computational approaches. Deep learning has recently demonstrated remarkable success in the field of SA in many languages including Arabic. Arabic sentiment analysis, however, still has to be improved, due to the complexity of the Arabic language’s structure, the variety of dialects, and the lack of lexicons. Moreover, in Arabic, anxiety as a psychological sentiment has not been the target of much research.

**Aim:**

This paper aims to provide solutions to one of the challenges of Arabic Sentiment Analysis (ASA) using a deep learning model focused on predicting the anxiety level during COVID-19 in Saudi Arabia.

**Methods:**

A psychological scale to determine the level of anxiety was built and validated. It was then used to create the Arabic Psychological Lexicon (AraPh) containing 138 different dialectical Arabic words that express anxiety, which was used to annotate our corpus (Aranxiety). Aranxiety comprises 955 Arabic tweets representing the level of user anxiety during COVID-19. Bi-GRU model with word embedding was then applied to analyze the sentiment of the tweets and to determine the anxiety level.

**Results:**

For SA, the applied model achieved 88% on accuracy, 89% on precision, 88% on recall, and 87% for F1. A majority of 77% of tweets presented no anxiety, whereas 17% represented mild anxiety and a mere 6% represented high anxiety.

**Conclusion:**

The proposed model can be used by the Saudi Ministry of Health and members of the research community to formulate solutions to increase psychological resiliency among the Saudi population.

## 1. Introduction

The World Health Organization reported a novel coronavirus disease in 2019, called COVID-19 and designated it a public health emergency of international concern that would pose a significant threat to humanity. In March 2020, the WHO announced that the disease could be characterized as a pandemic, which placed a great deal of pressure on populations around the world [World Health Organization (WHO), 2020]. This pandemic posed the greatest threat to human survival since World War II (Brahmi et al., 2020). In the early stages of the pandemic, when there was no vaccine, drug therapies or other aggressive treatments, the only protection from this deadly virus was social distancing and nationwide lockdowns. Almost all countries adopted a lockdown policy in order to minimize the spread of the virus (Barkur and Vibha, 2020). A psychological fear existed, because people did not understand the virus and it appeared to kill at random. Thus, people turned to social media to express their opinions based on their current states of mind and to communicate their feelings to friends and foes (Brahmi et al., 2020).

The coronavirus pandemic is expected to have a vast impact on the psychological effects of mental health. Mental disorders such as anxiety greatly contribute to the economic, social, and physical burden of people worldwide (Schwartz et al., 2014). Social media provides a means of self-expression and it facilitates measurement of the psychological status of those sharing their feelings (Kumar et al., 2019). Hence, social media data can be analyzed to provide solutions for psychological and mental health issues.

Sentiment Analysis (SA) is a technique to automate the classification of people’s sentiment attitudes, feelings or reviews employing natural language processing (NLP) and computational approaches. Recently, deep learning has achieved astounding achievements in the field of SA in several languages, including Arabic. However, because of the complexity of the structure of the Arabic language, the variety of dialects, and the scarcity of lexicons, Arabic sentiment analysis still has to be improved. Moreover, there has been very little investigation regarding the application of deep learning to Arabic sentiment analysis (ASA) related to anxiety.

This work attempts to determine the anxiety level of the Saudi population during the COVID-19 pandemic by applying ASA to Twitter data and providing solutions to one of the challenges of SA. To achieve this, the Arabic Psychological Lexicon (AraPh), comprising various dialectical Arabic words that express anxiety, was established. The lexicon was then used to annotate an Arabic dialectical corpus extracted from Twitter. In addition, the work evaluated the effectiveness of the application of the deep learning model. Bidirectional gated recurrent units model was applied to this work’s corpus because it achieved good accuracy when used to analyze SA of the Arabic dataset (Almuqren et al., 2019).

The main contributions of this paper are:

1. Define and investigating a psychological scale to determine the level of anxiety.
2. Identifying, based on the above scale and Twitter mining, the psychological effects of COVID-19.
3. Building an Arabic psychological Lexicon.
4. Analyzing the psychological situation of the Saudi community in the face of COVID-19.

The rest of the paper is organized as follows: Section 2 discusses related research. In Section 3, the design of the psychological scale is explained, followed by a review of the model construction. In section 4, the results and discussion are presented, and Section 6 concludes the paper.

## 2. Related research

### 2.1. The psychological influence of COVID-19

The emergence of COVID-19 has greatly disrupted people’s lives and livelihoods. It has led to restrictions on public transportation, a fear of viral transmission, school closures and work disruption. This has had devastating mental health effects, such as stress and anxiety (Fardin, 2020). Research conducted by Ozamiz-etxebarria et al. (2020) showed a positive correlation between the pandemic and high stress and anxiety levels, which were measured in a sample of 976 adults, using the Depression, Anxiety, and Stress Scale. The study also detected higher levels of stress and anxiety symptoms after the stay-at-home order was issued.

Salari et al. (2020) conducted a systematic review and meta-analysis of studies that focused on stress and anxiety prevalence among the general population during the COVID-19 pandemic in the Science Direct, Embase, Scopus, PubMed, Web of Science (ISI) and Google Scholar databases. The prevalence of stress was reported in five studies with a total sample size of 9,074, and the prevalence of anxiety was reported in 17 studies with a sample size of 63,439. The study concludes that COVID-19 not only causes physical health concerns, but also results in a number of psychological disorders.

### 2.2. Psychological changes under the action of social media

Liu and Liu’s (2020) study indicated that exposure to social media during the COVID-19 pandemic led to higher levels of anxiety and stress. The study was conducted with a sample of 1,118 Chinese subjects from 30 Chinese provinces. The findings showed that all four types of mass media (official, commercial, social, and foreign media) caused indirect trauma to audiences, hence the higher levels of anxiety and stress.

Zakout et al.’s (2020) study showed that COVID-19 has had serious consequences in many aspects of life, including negative psychological effects. The study aimed to assess the effect of the intensive media coverage of the pandemic on mental health. Higher prevalence rates of depression, stress, and anxiety were reported in non-Saudi participants compared to Saudi ones. Over one half (55.8%) of the participants reported feeling that the extensive coverage of COVID-19 in mass and social media had led to higher levels of mental distress.

In a cross-sectional study, Chinese citizens aged ≥18 were invited to participate in online surveys from 13 January to 2 February 2019 for a rapid assessment. There were 4,872 participants from 31 provinces and autonomous regions in the study. Levels of anxiety were assessed by the Chinese version of the General Anxiety Disorder Assessment (GAD7). Multivariable logistic regressions were used to identify associations between social media exposure and mental health problems. More than 80% of the participants reported frequent exposure to social media and higher levels of anxiety (Spitzer et al., 2006).

### 2.3. Anxiety prediction

Anxiety can be defined as “a complex emotional state that represents a mixture of feelings like constant fear, terror, dread, restlessness, and apprehension as a result of expecting an eminent negative future event or feeling threatened by a significant yet mysterious event that one cannot identify objectively.” The term refers to “any idea, situation, or event that gives rein to anger, nervousness or frustration”.

Data from various online platforms have been collected in previous studies to predict mental illnesses such as anxiety disorders, using a variety of approaches. The approaches used for the prediction of anxiety include using self-assessment questionnaires, self-declaration of diagnosis, membership of specialized online forums and manually annotated posts.

In the early literature, the main technique employed was to compare a participant’s self-reported social anxiety level with the result from raters based on objective criteria (e.g., number of interests, number of status updates) to determine the participant’s level of social anxiety (Fernandez et al., 2012). To facilitate automatic anxiety prediction, self-assessment questionnaires are also used, along with participants’ online behaviors, as in Zaman et al. (2020). The study aimed to identify individuals with anxiety and to estimate their levels of anxiety, personal online activity histories from YouTube and Google Search were gathered, as well as the participants’ clinically validated ground-truth anxiety assessment scores. A clinically validated questionnaire, GAD-7 (Spitzer et al., 2006), was used in the assessment.

Depression and anxiety traits were detected by looking at the images that Twitter users post and set as their profile pictures, as well as by surveys reporting on depression and anxiety, in a study by Guntuku et al. (2019). Image posting and profile picture preferences were analyzed by Guntuku et al. (2019) by employing image tag clusters, colors and aesthetic and facial features. This allowed the researchers to identify the way and extent to which these images revealed users’ mental health conditions.

The public posts of users who self-disclose about their anxiety disorders, as well as their online social network behavior and interaction characteristics, were examined in a study by Dutta and De Choudhury (2020). A supervised learning-based classifier identified those at risk of or experiencing anxiety, as self-reported on the Twitter platform, by studying users’ online social networks, interactions and social behaviors.

Spaces for discussion, asking for advice or receiving emotional support for topics related to mental health issues, like mental health communities’ forums, have provided data for certain studies. One example is ‘Detecting anxiety on Reddit’ (Shen and Rudzicz, 2017), which provided rich bodies of text from users in the context of self-assembled communities. Lexicon-based features [Linguistic Inquiry and Word Count (LIWC)] with n-gram probability were used in anxiety-related subreddit posts related to binary levels of anxiety.

To predict anxiety, other studies have used the annotation approach. For example, in Lee et al. (2019), a classifier was developed to detect whether tweets contained anxious content or not, based on an annotated dataset. In order to reflect comprehensive unpleasant feelings that may be related to social events, issues and atmospheres, this annotation was based on 18 emotions: nervousness, perplexity, worry, excitement, restlessness, frustration, apprehension, discomfort, fear, turmoil, yearning, depression, gloom, hostility, desperation, dismay, petulance and malaise (Lee et al., 2019). The degree of tweet anxiety was also estimated based on spatio-temporal features.

It is useful to observe the endeavors of studies identifying the level of anxiety during emergencies. The psychological reactions (anxiety) of Twitter users to the 2018 Hawaii false ballistic missile alert were explored by Jones and Silver (2020) by analyzing Twitter data before and after the event. A list of 114 anxiety words (e.g., afraid, scared, worried) available in the LIWC program were compared to the words in each tweet. The study found a distinct increase in anxiety among Hawaiian residents that remained long after the missile threat had been dismissed.

The psychological fear and anxiety among Twitter users caused by COVID-19 was demonstrated by the prevalence of negative emotions in tweets in a study by Singh et al. (2020). Tweets were collected for 3 days using the keywords CORONAVIRUS and COVID-19, and then analyzed with e-motion analysis. E-motion analysis examines eight emotions, which are classified into two groups; one group is related to negative sentiments (anger, disgust, fear and sadness) and the other to positive sentiments (anticipation, joy, surprise and trust).

It must be noted that the guidelines followed by annotators and content analysis for identifying symptoms of anxiety are derived from the researchers’ perspectives. Word emotions related to anxiety are defined by the authors (e.g., Lee et al., 2019; Singh et al., 2020). Moreover, the magnitude of anxiety is assumed to be distributed continuously rather than in a dichotomous fashion, and studies of anxiety prediction focus on discrete binary classes. They focus on the presence or absence of anxiety, rather than on the degree.

As far as the current authors are aware, the creation of a corpus (ground truth) for the analysis of anxiety, based on psychological assessment, and focusing on Arabic posts, has not yet been attempted. A variety of Arabic corpora have been created for natural language processing (NLP), for example by Almuqren and Cristea (2021), but none have targeted anxiety issues. This study has collected data to support the prediction of anxiety levels.

## 3. Methodology

### 3.1. Psychological scale design

One of the authors built a psychological scale for measuring the psychological effects of COVID-19, based on a literature review and her experience in the field. After that, it was reviewed by 10 experts in the same field to verify its effectiveness. The scale includes 20 statements that express the anxiety level of the participants. A 3-point Likert scale was used to record the level of anxiety during the COVID-19 pandemic. On the scale, 1 (Never) indicates no anxiety, 2 (Sometimes) indicates mild anxiety, and 3 (A lot/always) indicates moderate to strong anxiety.

#### 3.1.1. Evaluating the psychological scale

In order to evaluate the psychological scale, 20 tweets that mentioned the desired hashtags (explained below in Section 3.2) were randomly collected. Then, the deep learning model (Section 3.5) was applied to define the anxiety level for each tweet. In addition, a questionnaire for the psychological scale we developed (Section 3.1) was distributed to the authors of the analyzed tweets. The actual anxiety level derived from the questionnaire and the predicted level obtained by mining the tweets were compared using the deep learning model, see Table 1. The results proved the effectiveness of our psychological scale for use in this study in building our lexicon.

**Table 1:** Comparison of the actual and predicted anxiety level.

Label	Predicted	Actual
A (None)	47.62%	38.10%
B (Mild)	33.33%	28.57%
C (Moderate/Strong)	19.52%	23.81%

#### 3.1.2. Ethical approval

Every project influences human interests through legal, ethical, social and professional impacts. Project ethics, as defined in Cohen et al. (2011), are ethical rules that should be followed during a project for several reasons; some of them also affect a project’s validity and reliability. These rules include that, prior to the data collection stage, the researcher must obtain authorization from the target sample, the privacy and security of the participants’ information must be ensured, and questionnaire information must be saved and stored in a secure place. To avoid any legal and ethical issues, this process was followed, and the ethical form was submitted to the Institutional Review Board at Princess Nourah bint Abdulrahman University (Table 2).

**Table 2:** Anxiety level and the total number of unique tweets in *Aranxiety.*

Anxiety Level	# of unique tweets
A (None)	740
B (Mild)	160
C (Moderate/Strong)	55
Total	955

### 3.2. The *Aranxiety* dataset

We named our dataset the *Aranxiety Dataset*. To build it, we used Python to fetch Arabic tweets originated from Saudi Arabia based on certain search terms. To collect the relevant tweets, we extracted the relevant top hashtags that mentioned COVID-19 and quarantine in Arabic. As result, top keywords such as: #المنزلي_الحجر, #فعاليات_الحجر_المنزلي, الحجر المنزلي in English are: #home_quarantine, #home_quarantine_activites, home quarantine were used as keywords in a search query to fetch tweets from Twitter. We gathered tweets continuously from April to June 2020, mainly because this period marked the beginning of the COVID-19 pandemic in Saudi Arabia. Our corpus Aranxiety comprises 955 Arabic tweets representing the user anxiety level during COVID-19.

To clean the *Aranxiety* corpus, non-Arabic tweets were eliminated, and re-tweets were discarded. All features that were unnecessary and would decrease the classifier’s accuracy, e.g., links, user mentions, punctuation marks and stop words, were filtered out. Pre-processing was applied to the dataset (tokenization and normalization). Normalization involved, for example, removing *kashida* (expanding letters) and uniting the same letters with different shapes. The cleaning and pre-processing were done using Toolkit (NLTK) library in Python. Examples from before and after pre-processing are shown in Table 3.

**Table 3:** Subset of the *Aranxiety* corpus before and after pre-processing.

Tweet before pre-processing	Tweet after pre-processing	Anxiety level	Tweet in English
تسجيل اول حالة في العلا الوضع أصبح مرعب جداً والمرعب أكثر ان منطقتي صغيرة #خليك_بالبيت #وزارة_الصحة #كارونا_السعودية #حظر_التجول_في_السعودية #الحجر_المنزلي #كوفيد_19	تسجيل اول حاله في العلا الوضع أصبح مرعب جدا والمرعب اكثر ان منطقتي صغيره	C	The first case was recorded in Al-Ula. The situation has become very terrifying, and it is even more terrifying that my area is small.

### 3.3. AraPh anxiety lexicon

The Arabic Psychological Lexicon (AraPh) was built by one of the authors based on the 14 anxiety trait axes. Every axis is expressed by different words in the various Arabic dialects, with 17–18 different words for each axis. Modern Standard Arabic (MSA) words were not considered, as MSA is the dialect used for example by academics, linguists and the media, and it is not often used in Twitter chat. The lexicon contains 138 different dialectical Arabic words that express anxiety, see Table 4. The lexicon has been assessed by 10 experts in the field, see Appendix 1. Finally, the authors (L and F) used the AraPh lexicon as a guide to annotate the corpus.

**Table 4:** AraPh lexicon statistics.

Anxiety trait axes	Number of words
Stress	11
Instability	9
Nervousness	18
Unhappiness	10
Failure	8
Uncomfortable	7
Accumulation of problems or business	11
Disturbing thoughts	8
Lack of self-confidence	10
Difficulty making decisions	10
Feelings of inadequacy	8
Dissatisfaction	9
Rapid impact	10

#### 3.3.1. Annotation of *Aranxiety* corpora

A sentiment label was added to the dataset. We used the trinary labels (*A*, *B*, *C*) to annotate the dataset, where *A* denotes no anxiety, *B* represents mild anxiety and *C* indicates moderate to strong anxiety. Each label expresses the anxiety level in each tweet, following previous recommendations (Al-twarish, 2016). Although a given tweet might have other emotions associated with it, we discard these for the time being. Liu (2012) mentioned two techniques for building a lexicon: automatic and manual techniques. Automatic techniques include two approaches: the dictionary-based approach and the corpus-based approach (Liu, 2012). The manual approach is time and labor consuming but more accurate than the automatic approach (Al-Twairesh, 2016). Therefore, the annotation process was carried out manually for the *Aranxiety* corpus by three annotators (computer graduates) who had experience of the annotation process. Every annotator needed to assign one label per tweet for the whole corpus. Before we began the annotation process, the annotators were provided with annotation guidelines in Arabic as the annotators were native Arabic speakers. Three annotators, instead of the usual two, are used to identify the annotation scheme’s reliability. To increase the quality of the resulting corpus by alleviating conflicts that could arise from discrepancies between only two annotators. Hence, if two annotators disagreed concerning one tweet classification, we voted between all three annotators. Some of the annotation guidelines are shown in Appendix 2. We stored the annotations in an Excel file, see Appendix 3.

### 3.4. Evaluation metrics

To evaluate the performance of the models, we used four metrics suitable for classification (Chicco and Jurman, 2020): the micro averages of precision, recall, F1 and accuracy. The micro average totals the contribution of all classes to the average metric calculation (Sokolova and Lapalme, 2009). It aggregates the precision and recall of the classes.

### 3.5. Deep learning model construction

The most popular deep learning-based model, GRU, was used in this study. GRU is variant of Recurrent Neural Network (RNN). We used a bidirectional GRU bi-GRU rather than other deep learning models, because prior research has shown bi-GRU to provide high accuracy for Arabic sentiment analysis (Almuqren et al., 2019). Keras (Chollet, 2018) was used for the deep learning models. In addition, TensorFlow (Abadi et al., 2016), an open-source library, was used in a Graphics Processing Unit (GPU) environment. Two embeddings were utilized to obtain the features: character-level and Word2Vec. In Word2Vec, the features were obtained using word representations to expose the connections between the words in the tweets. Character-level was used to show how the sentiment affects the different characters in the tweets.

**Figure 1:**
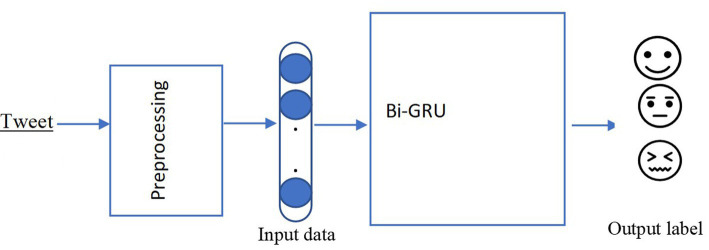
Deep learning architecture.

The model started with word embedding, to represent each word in a tweet as a 300-dimensional word vector. It was then fed into the GRU layer with this embedding, using a 128-dimensional hidden state. To avoid the model overfitting through training dropout (Xiong et al., 2019), the output was fed into another GRU layer with a 128-dimensional hidden state that returned a single hidden state (Figure 1). Different experiments were done on 20, 40, 50, 70 and 100 epochs, where the number of epochs is the number of complete passes through the training dataset. The best performance was accomplished at 50 epochs. Therefore, all the reported experiments were conducted with 50 epochs. The sigmoid layer was used for the classification. We applied a dense layer with 2 units for the two possible classes, followed by the sigmoid activation. In addition, we used backpropagation in a default implementation bundle with the TensorFlow library. For optimization of the weight, Adam (Kingma and Ba, 2015) was used, because it has been shown to be efficient in computation.

## 4. Results and discussion

### 4.1. Model results

The *Aranxiety* corpus was split into 20% for testing and 80% for training; additionally, 10-fold cross-validation was performed on both to obtain the best error estimate (James et al., 2013). 10-fold cross-validation is used to validate the performance of a classifier. The fundamental concept of cross-validation is to split the original dataset into two parts: the training set and the testing set. Train the classifier using the training set and test the model using the testing set to review the classifier’s performance. To counter oversampling due to the dataset being biased toward negative tweets, we used the popular Synthetic Minority Over-Sampling Technique (SMOTE).

Table 5 shows that the results of our model had 88.0% accuracy and the F1 is 87%. Adding the bi-directional model attention mechanism enhanced the model’s performance.

**Table 5:** Model results.

Label	Precision	Recall	F1	Average
A	87.0	99.0	92.0	
B	94.0	57.0	71.0		
C	88.0	78.0	82.0		
Accuracy						88.0
Macro avg	90.0	78.0	82.0	
Weighted avg	89.0	88.0	87.0		

After analyzing the tweets, we found that the majority showed no indication of anxiety due to COVID-19, which was surprising for us. The word “nervous” was mentioned more than any other in the tweets, and this may be due to people’s nature and a wish to pretend to be positive on social media. In addition, it may be due to the conservative nature of Saudi community.

### 4.2. Discussion

During the COVID-19 pandemic, with the quintern, people used social media to release their sentiments. Therefore, social media generates significant data; we can use it to provide mental health and psychological solutions. Our study aimed to mine Saudi Arabian tweets using a deep learning model to measure the anxiety level during COVID-19 in Saudi Arabia.

First, we built and arbitrated a psychological scale to determine the level of anxiety. To be sure that the scale is valid for use, we did a pilot study with 20 persons chosen randomly from Twitter. We mined their tweet using our proposed model to measure their anxiety. Besides, the scale was sent for them to measure their anxiety levels. We found that the results from scale and Twitter mining are similar, meaning that mining tweets are a valid tool to measure and predict people’s anxiety level, and the scale is valid.

It has built the first Arabic Psychological Lexicon (AraPh) containing 138 different dialectical Arabic words that express anxiety. 10 psychological experts assessed it. Then, we used the lexicon to annotate our corpus. Our corpus Aranxiety comprises 955 Arabic tweets representing the user anxiety level during COVID-19.

The proposed multi-way (Three-way) model was applied to the Arabic corpus; the multi-way SA model is complex for machine learning to predict (Al Shboul et al., 2015). We applied bi-GRU with word embedding that approved the high performance with Arabic dialect corpus before (Almuqren et al., 2019) on our dataset. The bi-GRU model achieves 88% accuracy. The F1 score for the *A* label, which refers to no anxiety, is much higher than for the *B* label, which refers to mild *anxiety* and *the C* label, which refers to moderate/ strong anxiety labels. This may be due to unbalancing in the dataset. As we explained before to avoid the overfitting, we applied many techniques.

As far as we know, creating a corpus (ground truth) for analyzing anxiety based on psychological assessment, and focusing on Arabic posts, has not been attempted before. Various Arabic corpora have been created for NLP, yet none target anxiety issues. This study collected data to support the prediction of anxiety levels during the COVID-19 pandemic. The surprising result is that the majority had no anxiety – a possible explanation for this may be that this was due to the control exercised by the Saudi Health Ministry during the pandemic.

### 4.3. Comparison and implication

By using our model on SemEval’s Arabic data set from their 2017 Task 4, Subtask A, which classifies tweets based on a three-point scale, our proposed model achieved an accuracy of 80.7%. This is a significant improvement compared to the NileTMRG team’s accuracy of 58.1%, who were ranked first among the top 10 teams in Subtask A. This shows promising progress in Automatic Sentiment Analysis (ASA) on tweets.

## 5. Limitation

One of the limitations of this work is that the dataset used for the classification has unbalanced classes. The majority class with no anxiety represents 77%, mild anxiety represents 17%, and only a small percentage (6%) represents high anxiety. One potential reason for this is that Saudi society is conservative, and people tend not to express their anxiety on social media such as Twitter. The biggest challenge in this research was collecting data that fit the specified criteria.

## 6. Conclusion

This research was conducted to assess the psychological effects of a global pandemic based on Arabic tweets. Our research mined Saudi tweets using a deep learning model to analyze the psychological effects of COVID-19 in Saudi Arabia. This study resulted in the construction of an anxiety corpus of Saudi tweets related to COVID-19, consisting of 955 tweets. Bi-GRU (88% accuracy) was applied and interestingly and contrary to our initial expectations, the results showed that the majority of tweets contained no indications of anxiety, although there are a great number of mentions of “nervous” in the Arabic tweet corpus. Future work could use a larger dataset with more balanced classes. Future research is also needed to test GRU models with different implementations and to test more features to potentially further raise the accuracy. Future work could use a larger dataset using various types of search keywords to improve the diversity of tweets and with more balanced classes.

## Data availability statement

The raw data supporting the conclusions of this article will be made available by the authors, without undue reservation.

## Author contributions

MA: psychological analysis. DP: review the psychological analysis. FA: collect the data. LA: technical analysis. AC: review the technical analysis. All authors contributed to the article and approved the submitted version.

## Conflict of interest

The authors declare that the research was conducted in the absence of any commercial or financial relationships that could be construed as a potential conflict of interest.

## Publisher’s note

All claims expressed in this article are solely those of the authors and do not necessarily represent those of their affiliated organizations, or those of the publisher, the editors and the reviewers. Any product that may be evaluated in this article, or claim that may be made by its manufacturer, is not guaranteed or endorsed by the publisher.
